# Is Health Contagious?—Based on Empirical Evidence From China Family Panel Studies' Data

**DOI:** 10.3389/fpubh.2021.691746

**Published:** 2021-07-02

**Authors:** Feng Hu, Xiaojiao Shi, Haiyan Wang, Nan Nan, Kui Wang, Shaobin Wei, Zhao Li, Shanshan Jiang, Hao Hu, Shuang Zhao

**Affiliations:** ^1^Global Value Chain Research Center, Zhejiang Gongshang University, Hangzhou, China; ^2^School of Economics, East China Normal University, Shanghai, China; ^3^School of Economics and Management, Shihezi University, Shihezi, China; ^4^School of Economics, Huazhong University of Science and Technology, Wuhan, China; ^5^International Business Research Institute, Zhejiang Gongshang University, Hangzhou, China; ^6^School of Master of Business Administration, Zhejiang Gongshang University, Hangzhou, China; ^7^School of Economics, Shanghai University, Shanghai, China

**Keywords:** health, contagion, health in China, self-rated health, contagion mechanism, endogeneity control

## Abstract

This study empirically analysed the contagion of health using data from China Family Panel Studies. We first controlled variables related to health behaviour, medical conditions, individual characteristics, household characteristics, group characteristics, and prefecture/county characteristics and then employed multiple methods for estimation. The estimates showed that the average health level of others in the community had a significant positive effect on individual self-rated health—health was contagious. The measurement results remained robust after the endogeneity of the core explanatory variables was controlled using two-stage least squares. Furthermore, by analysing the heterogeneity of health contagion, we found that the contagion effect of health varied with the level of medical care, household affiliation, gender, rural/urban areas, and age groups. The contagion effect of health was more pronounced in the elderly population and the rural areas of the central region, where the level of medical care is relatively low, whereas it did not differ significantly between genders. Finally, the learning or imitation mechanism and social interaction mechanism of health contagion were examined.

## Introduction

Medical and health services are major issues related to people's health and families' well-being. As an important component of human capital, health is an inevitable requirement for promoting comprehensive human development and a basic condition for economic and social development. In particular, the impact of the COVID-19 outbreak in late 2019 has increased people's awareness of the importance of health and their concern about health issues. Presently, China is vigorously promoting the strategy of constructing a “Healthy China” and has promulgated the “Healthy China 2030” blueprint, which aims to improve the health of all Chinese people by raising the importance of health in the development strategy and prioritising the improvement of people's health. However, it is also necessary to be aware that the current gap between people's need for a healthy life and the inadequate and imbalanced development of medical and health services remains prominent, which has hindered the improvement of people's health to some extent. Given these circumstances, it is theoretically and practically significant to explore the issue of health contagion.

Numerous factors influence health. For example, many existing studies have analysed the impact of demographic characteristics and socioeconomic factors on health. Regarding demographic characteristics, Banister and Zhang (1) found a gap between female and male health. Similarly, Wagstaff et al. (2) used Chinese Longitudinal Healthy Longevity Survey (CLHLS) data and found that although the health level of the elderly in China improved between 1998 and 2008, health inequalities widened after this period (with the worst health status being seen among the elderly in western regions, the elderly in rural areas, female elderly, and elderly people without spouses). Van Doorslaer and Gerdtham (3) confirmed a significant negative relationship between age and health. In addition, marital status has an impact on individuals' health. Regarding this, Gardner and Oswald (4) argued that marriage could improve individuals' health for two possible reasons. First, married people are inherently healthier than unmarried ones; second, single people are more likely to develop bad habits and be more socially separated than married ones. In addition, Preston (5) found that an increase in income can promote individual health levels; however, the positive effect of absolute income on health decreases as income levels grow. Kools and Knoef (6) argue that only permanent income can affect health levels, and absolute income directly improves individual health levels while also having an indirect impact (7). Grossman (8), Conti et al. (9), and Mueller et al. (10) found that education improves people's health. Additionally, Muurinen (11) put forth the theory of health depreciation, and Fujishiro et al. (12) concluded that occupation is positively associated with self-rated health based on data from the U.S.

In addition, health investment is an important factor that affects health. The impact of health investment on individual health has continued to spike the interest of researchers in China and worldwide. Perrin and Valvona (13) found that increased health investment improves people's health. McClellan et al. (14) and Barnato et al. (15) concluded that intensive treatment resulting from increased health investments significantly reduces people's mortality levels. In developing countries, efficiency and equity may be more important than the scale of government health investment. Zhai et al. (16) found that governments' public health investment could improve the health status of people, and this effect is more obvious for inland areas than coastal provinces, which was also verified by Jiang et al. (17), Zhai et al. (18), and Gallet and Doucouliagos (19). However, Newhouse and Friedlander (20), Wolfe (21), and Tanzi and Schuknecht (22) found that the effects of government health investments are not significant. Fisher et al. (23) also concluded that regions spending more on medical insurance coverage do not achieve better health outcomes than those spending less on the same. Similar results were reported by Chinese researchers in their studies (24). In addition to studying the impacts of health investment on individual health, researchers have also analysed how various social benefits are formed based on health investment influence. Among researchers outside China, Young and Cohen (25) found that patients without medical insurance have a significantly higher post-discharge mortality rate than those with medical insurance. Cohen (26) also concluded that patients without medical insurance are diagnosed with diseases later and have a lower survival rate after curing.

The previously mentioned studies have viewed the health level of an individual as being independent of that of others in the group. However, several studies have found that individuals' behaviour, thoughts, and emotions are easily influenced by the thoughts and behaviours of others (27–30). For example, if the happiness score of an individual's neighbours and friends living nearby increases by one unit, the individual's happiness will increase by 25–34% (31). Further, if the average consumption level of an individual's roommates increases by CNY100, the individual's consumption expenditure will increase by CNY21.5 (32). Researchers often refer to the effects of other people's behaviour, thoughts, or emotions on individual behaviour as neighbourhood effects. Based on this, it is worth asking whether an individual's health—an important indicator of individual well-being—is also influenced by other members of a group. In other words, do neighbourhood effects result in an improvement in the health and well-being for individuals? To date, little research has been conducted to demonstrate the neighbourhood effects of health and its formation mechanism. In fact, in a typical “relational” society like China, the thoughts and behaviour of Chinese residents are easily influenced by those of others due to the strong notion of collectivism. Therefore, we hypothesised that an individual's health level would also be influenced by other members of the group.

Sociologists believe that health behaviours are an intermediate mechanism of health transmission. According to the impact of health behaviours on personal health, individuals are divided into two categories. One category promotes health behaviours, such as regular exercise, intake of a balanced diet, adherence to medications, and obtaining health knowledge. The other encourages health-hazardous behaviours, such as smoking, drinking, staying up late, lack of exercise, and drug abuse. Specifically, we believe that there are two main mechanisms of healthy transmission: learning or imitating mechanisms. Individuals learn or imitate the good health behaviours of the people around them or are imperceptibly influenced by those around them to produce the same or similar health behaviours and then improve their health conditions. For example, other people have the reasonable habit of running, exercising, and collocating nutrition, which may induce us to rationally exercise, run, and arrange nutrition every day. This is a learning or imitation mechanism. This is an active health behaviour. Individuals tend to actively strengthen their investment in health behaviours to improve their health. The second is the social interaction mechanism. Individuals enrich their health knowledge and improve their health behaviours through information exchanges and discussions with people around them, thereby promoting the improvement of their personal health. Conversely, based on trust and mutual benefit, community members can reach a cooperative network, cultivate others' sense of meaning and goal in life, enhance others' mental health, reduce the impact of stress on others' health, and guide individuals to engage in the behaviour of protecting others' health and their personal health.

The remainder of this paper is organised as follows. Section literature review reviews relevant literature, including studies on neighbourhood effects, peer effects, and health. Section materials and method introduces the research design, including the data sources, variable selection, and model setting. Section analysis of empirical results presents the analysis of empirical results, including the baseline model regression analysis, endogeneity control, robustness test, and heterogeneity analysis. Section analysis of empirical result describes the tests for the two mechanisms of health contagion—the learning or imitation mechanism and the social interaction mechanism. Finally, section limitations and future research dire concludes this paper.

## Literature Review

This study is mainly related to three types of literature. One type is regarding the neighbourhood effects. The literature outside China focuses on the impact of neighbourhood effects from four aspects: [1] The impact of neighbourhood effects on adult individuals' health, income, consumption, and poverty. Shouls et al. (33) found that if the poor live in remote areas, their health worsens. By developing a multilevel econometric model, Fang and Zou (34) confirmed that neighbourhood effects are significant in terms of individual poverty, and individuals living in neighbourhoods with higher rates of poverty are more likely to have low income and thus fall into poverty. By exploring whether school poverty is a mediator of neighbourhood effects on students' academic achievement, Wodtke and Parbst (35) found that the impact of school poverty on students' academic achievement is not significant during both childhood and adolescence. [3] Threshold and non-linear characteristics of neighbourhood effects influencing individual social behaviour. Using the unemployment rate as a neighbourhood characteristic, Buck (36) conducted a study using UK household panel data and found a significant non-linear relationship between the probability of unemployment and that of falling into poverty when the proportion of unemployed neighbours exceeds 23–24%. When the neighbourhood poverty rate exceeds 20%, it has a significant effect on individual delinquent behaviour and school dropout behaviour. [4] Impact of neighbourhood effects on individual adolescent behaviour. Nie et al. (37) used a study sample of Chinese children and adolescents (aged 3–18 years) to confirm the significant impact of neighbourhood effects on obesity. They also found that neighbourhood effects are stronger in rural areas, among individuals at the high end of the body mass index (BMI) distribution and among females.

Compared with foreign studies, studies in China remain in their infancy, as they mainly focus on individual and household behaviours, such as gambling participation, stock market participation, household donation activities, household education expenditures, residents' mental health, and individual poverty. Li and Zhou (38) argued that the neighbourhood effects of group members' participation in gambling can raise individuals' gambling participation expectations, while the irregularity of the gambling system reduces the positive role of neighbourhood effects. Many studies have found that neighbourhood effects significantly influence residential stock market participation. By extending the individual stock investment optimisation model, Wang et al. (39) found that family social networks (i.e., those based on ties with family and friends) increase the likelihood of residential stock market participation. Once residents enter the stock market, the shares of their stock holdings in their financial wealth increase. Zhou et al. (40) argued that neighbourhood effects can more significantly drive residents' stock market participation for communities with a high proportion of local goods expenditures and a highly concentrated income distribution. Similarly, Zhu et al. (41) argued that for every 1% increase in the neighbourhood effect indicator, the probability of household participation in the stock market increases by ~0.2%, and the degree of participation in the stock market increases by ~0.7%. However, neighbourhood effects have no significant impact on stock market returns. In addition, neighbourhood effects have a significant impact on household donation activities and household education expenditures. Yan et al. (42) concluded that for every 1% increase in the average social donation activity of community groups, the probability of households choosing social donation increases by approximately 0.327% to 0.549%. Other information access channels, such as the Internet, reduce the extent to which neighbourhood effects impact household social donation activities. Yu and Zhan (43) found that for every 1% increase in the average education expenditure of households in the community, household education expenditure increases by ~0.307%. For rural areas, the widening income gap between middle- and high-income households and between low- and middle-income households make household education expenditure more sensitive to the average education expenditure of other households in the same community. Yao and Jiang (44) found the existence of neighbourhood effects in farms' behaviour and concluded that neighbourhood effects among farms lead to a shift from non-safe production behaviour to safe production behaviour.

Other studies are related to peer effects. This topic has attracted considerable attention and extensive discussion among Western researchers, but relevant research has also been conducted in China. Research on peer effects has been conducted from the perspective that individual behaviours are transmitted through social relationships. However, such studies have mostly focused on a certain group and primarily examine the cluster effect in education and health, including the influence of peer groups on adolescents' academic achievement, violent behaviour, and reading habits, the influence of roommates in dormitories on college students' social activities, consumption behaviours, career choices, smoking and alcohol abuse, and the influence of peer groups on college graduates' intentions to start a business. For example, Victor and Analía (45) verified the existence of gender-based peer effects by studying students' academic achievement at three different academic levels (elementary, middle, and high school) in Israel. They found that the proportion of female students in a class positively contributed to the class's academic achievement and student-teacher relationships. Jane (46) used data from fourth and fifth graders in public elementary schools in North Carolina to verify that peer effects have a significant impact on academic achievement and that peer effects of lower-achieving students are greater than higher-achieving students. Moreover, the study shows that peer effects only work within the same race (e.g., White students vs. White students, non-White students vs. non-White students) and that peer effects are not significant across races. Daniel et al. (47) used a natural experiment with college roommate assignments to test whether peer effects exist in several behaviours that significantly affect health and well-being, such as alcohol abuse, smoking, illicit drug use, gambling, sexual behaviour, suicidal ideation, and non-suicidal self-harm. The results showed that peer effects exist in alcohol abuse and smoking and that the peer effects of smoking are significant among males but not females; no other evidence indicated the existence of peer effects for the other factors. Cheng (32) also confirmed that peer effects exist in college students' consumption behaviours (such that for every CNY100 increase in the average consumption level of college roommates, a student's consumption expenditure increases by CNY21.50). Dong and Qin (48) focused on 14 colleges and universities in Shaanxi and Chongqing to analyse the impact of peer groups on college graduates' willingness to start a business and the impact mechanism. The results; the study showed that college students have greater intentions to start a business when there are other entrepreneurs in the peer group; the closer the relationship with the entrepreneurs, the greater the effect. Zong and Li (49) found that the presence of bad peers in the class has a significant negative impact on students' academic performance and that the academic achievement of male students, students from poor families, and resident students is more sensitive to bad peers in the class.

The third type focuses on health and factors affecting individual health. Such studies are relatively well-established, especially empirical studies that examine factors affecting individual health in terms of individual micro characteristics and the macro environment. Micro characteristics mainly refer to individual and household characteristics—gender, age, work, educational level, cognitive ability, household wealth level, population size, marital status, and urban/rural affiliation. Studies related to micro characteristics focus on adolescent groups, such as college students and retired elderly people. The macro environment mainly refers to the macroeconomic conditions that affect individual health, such as the economic development level, medical care, fitness facilities, medical insurance, and Internet penetration ratio. Generally, men have better health levels than women; the age depreciation effect of health creates significant health differences among different age groups. Having a spouse can significantly improve the cognitive function and health level of individuals; education can influence individuals' perception of health and related health behaviours. Further, in particular, people with higher educational levels have higher cognitive ability and adaptability and better health knowledge, which tends to result in healthier lifestyles and behavioural choices (9, 50, 51). Internet use also contributes to improving individuals' health by increasing income levels and the likelihood of exercise (52, 53). Medical insurance for urban residents significantly contributes to health improvement among the elderly and low-income and less healthy individuals (54, 55). Individuals with low socioeconomic status are disadvantaged in terms of exposure to and mitigation of stress, longevity gains, risk perceptions, and complementary health behaviours and are more likely to engage in risky health behaviours. However, members of upper social classes are less likely to engage in risky health behaviours and tend to lead healthier lifestyles (56–58).

Although the abovementioned research explored neighbourhood effects, peer effects, and residents' health, it is generally limited to a certain aspect of research, and few studies have been conducted on health contagion. In addition, this literature is only targeted at a certain group of people (e.g., children and adolescents such as students or retired elderly people), thereby failing to consider residents' health in a holistic manner. Furthermore, studies have focused more on subjective health indicators, such as sleep, anxiety, and depression, and lacked comprehensive indicators reflecting individual health status, making it difficult to grasp the relationship between individual health and group health.

Based on this analysis, there is still room for discussion on health contagion. Furthermore, it would be theoretically and practically significant to scientifically summarise whether the individual health of Chinese residents is influenced by group health and the influencing mechanism. In this context, we used the 2018 China Family Panel Studies (CFPS) microdata from the Institute of Social Science Survey (ISSS) of Peking University to systematically analyse the contagion of health.

## Materials and Methods

### Data Sources

This study mainly used the 2018 CFPS microdata from the ISSS of Peking University. The Chinese Social Science Research Center of Peking University was established in September of 2006. It is a data survey platform for Peking University's social sciences and an interdisciplinary platform for Peking University to conduct empirical research on social issues in China. The data currently released by the centre include the CFPS and CHARLS data, and the article uses CFPS 2018 data. Data at the individual, household, and community levels are biennially collected to reflect the changes in China's economy, society, and other aspects. The CFPS focuses on the economic and non-economic welfare of Chinese residents and many research topics, including economic activities, educational achievements, family relations and family dynamics, population migration, and health. It is a national, large-scale, and multi-disciplinary social tracking survey project.

The CFPS2018 sample covered a total of 25 provinces, municipalities, and autonomous regions, with a sample size of ~16,000 households in each period. The database contains information on all members of each household and is considered as representative nationwide household survey data. There are four types of questionnaires included within it: one each for communities, households, adults, and children. We mainly used questionnaires for communities, households, and adults for this study. As the three types of questionnaires involve different levels of data, we merged the data using Stata 15. After missing key variable values and invalid individuals were excluded, a sample of 20,371 individuals, 7,735 households, and 1,667 communities were used.

### Variable Selection

#### Explained Variable: Individual Self-Rated Health

There are usually two methods to measure international health levels. One is the self-rating of health level method, which measures the entire health status of a respondent through one question; the other is the comprehensive evaluation method, which simultaneously examines multiple dimensions of health and combines the information of each dimension to generate a comprehensive indicator. In this study, we used the self-rated health level method for the follow-up analysis, as it has good reliability and validity and provides a better picture of people's overall health status compared to objective indicators of disease status (59, 60). Self-rated health was measured by the question “How healthy do you think you are now?” The answers were given on a scale of 1–5, ranging from “unhealthy,” “fairly unhealthy,” “relatively healthy,” “healthy,” and “very healthy” (i.e., the higher the rating, the better the health).

#### Core Explanatory Variable: Average Health Level of the Community

An important core explanatory variable in this study was the health level of others. Given that health contagion is closely related to spatial distance (61–63), we need to specify the range of groups to which households belong when constructing this variable. This range should not be too large or too small. The CFPS2018 survey locations were divided into four levels: province/autonomous region/municipality, county/prefecture, town/street, and neighbourhood/village committee. The interview locations for the survey data are specific to neighbourhood committees, which provide the right locational space to explore the contagion of health in this study. Residents within the same community (including both urban neighbourhood committees and rural villages) meet each other frequently, and each community has a common activity space and activity centre. Within a community, residents are closely connected to each other, and their interactions form a social network. Based on this, we classified households from the same community as one group. In accordance with Nie et al. (37), we adopted the common method of calculating the average health level of others (except the respondent) in the existing literature and defined the average health level based on that of other members of the community (excluding the respondent). The calculation method is expressed as shown in [1].

(1) $$\begin{array}{l} \bar{{Health}_{j}}=\frac{1}{N-1}\overset{N-1}{\underset{j\neq i}{\sum }}\left(Health_{j}\right)\; \end{array}$$

where N represents the number of respondents in the community.

### Control Variables

Referring to the existing literature, we also added other factors that influence the health status of individuals, as these variables may reduce the omitted variables to some extent. These variables include individual characteristics, household characteristics, group characteristics, health behaviour, and the level of medical conditions. Individual characteristic variables included gender (a 0/1 variable; 1 = male), age, squared term of age, marital status (a 0/1 variable; 1 = married), educational level, and whether the Internet is accessible (a 0/1 variable; 1 = yes). In general, individuals who are married, those with access to the Internet, and those with higher educational levels are more likely to manage their health and have better general health. The Internet also has a positive impact on the health of the residents because Internet use can improve the efficiency of health investment, save medical costs, and obtain better health knowledge and information. Household characteristic variables included household size, household income level, and whether the household is in an urban or rural area (a 0/1 variable; 1 = urban). Community characteristic variables included the average age, average household income, average gender, and average educational level in a community, which can be calculated using Equation (1). Health behaviour variables included smoking (a 0/1 variable; 1 = yes), the number of cigarettes smoked per day, the frequency of exercise^1^, whether there had been alcohol abuse in the past month, type of medical insurance,^2^ and duration of sleep. Variables that reflect local medical conditions include the level of medical care at the point of visit, satisfaction with medical care conditions^3^, and the grade of the hospital visited. Other control variables included a dummy variable for the prefecture or county. The descriptive statistics for the specific variables are presented in Table 1.

**Table 1:** Definition and descriptive statistics of variables.

**Variable type**	**Variable name**	**Variable symbol**	**Sample size**	**Average value**	**Standard deviation**	**Minimum value**	**Maximum value**
Explained variable	Individual self-rated health	Health	20371	2.9748	1.2069	1	5
Core explanatory variables	Average health level of the community	avg_health	20371	2.9748	0.5401	1	5
Health behaviour variables	Whether to smoke	Smoking	20371	0.2814	0.4497	0	1
	Number of cigarettes smoked per day	smoking_s	20371	4.1523	8.3206	0	100
	Frequency of exercise	Exercise	20371	5.4523	2.7987	1	50
	Alcohol abuse in the past month	Drinking	20371	0.1467	0.3538	0	1
	Type of medical insurance	Insurance	20371	9.834	19.843	1	5
	Length of sleep	Sleep	20371	1.9508	3.3384	0	20
Medical condition variables	Level of medical care at the time of visit	medical_level	20371	3.5170	0.8797	1	5
	Satisfaction with medical care conditions	medical_ satisfaction	20371	3.6420	0.8185	1	5
	Grade of hospital visited	medical_place	20371	2.7456	1.5885	1	5
Individual characteristic variables	Gender	Gender	20371	0.5142	0.4998	0	1
	Age	Age	20371	48.870	16.864	16	95
	Squared term of age	age2	20371	2672.658	1627.879	256	9,025
	Marital status	Marry	20371	2.1292	0.8924	1	5
	Educational level	Edu	20371	2.9316	1.3989	1	8
	Access to mobile Internet	Mobile	20371	0.5228	0.4995	0	1
Household characteristic variables	Household size	fml_count	20371	4.2501	2.1321	1	17
	Household income level	Lnfinc	20371	10.682	1.335	0	14.457
	Urban or rural area to which a household belongs	urban	20371	0.5220	0.4995	0	1
Community characteristic variables	Average age in the community	avg_age	20371	48.249	10.382	0	93
	Average household income in the community	avg_lnfinc	20371	10.803	1.5755	0	13.415
	Average gender in the community	avg_gender	20371	0.5192	0.2094	0	1
	Average educational level in the community	avg_edu	20371	2.8792	0.8781	0	7

*Source: The data were obtained based on the CFPS2018 data from the ISSS of Peking University*.

### Statistical Analysis

To analyse the impact of community average health on individual self-evaluation health, we conducted the following two analyses. First, descriptive statistics of all variables are provided, including sample size, mean value, standard deviation, maximum value, and minimum value of variables. Second, the influence of community average health level on individual self-evaluation health was tested using regression analysis. In the regression, other factors influencing individual health status were added, including individual characteristic variables, family characteristic variables, group characteristic variables, health behaviour variables, medical condition levels, and other control variables. All regressions were performed using a stata/mp 15.0.

## Model Settings

### Setting the Baseline Model

Based on the characteristics of the CFPS2018 cross-sectional data, the following baseline model was constructed to verify the effect of the average health level in the community on individual health:

(2) $$\begin{array}{l} Health_{i}\;={\;\alpha }_{1}\;+{\;\alpha }_{2}\;\bar{{Health}_{i}}\;+{\;\alpha }_{3}{\;X}_{i}\;+{\;\mu }_{v}\;+{\;\varepsilon }_{i} \end{array}$$

where *Health*_*i*_ denotes the individual health status; $\bar{{\;Health}_{i}}$ is the average health level of the community; *X*_(*i*)_ represents the control variables, including health behaviour variables, medical condition variables, individual characteristic variables, household characteristic variables, and group characteristic variables; μ_*v*_ is a dummy variable for prefecture or county; and ε_*i*_ is the random disturbance term.

### Setting the Mediating Effect Model

To effectively reveal the transmission mechanism of the impact of average health levels in a community on individual health levels, we set the following recursive model to test the effect of the mediating variable, which is based on the testing method proposed by Zarit et al. (64): : ➀ the effect of the average health level in the community on individual self-rated health was tested; if the coefficient of the average health level in the community was significant, it indicated that the average health level in a community had a significant effect on individuals' self-rated health; ➁ the effect of an increase in the average health level of the community on the mediating variable was tested; if the coefficient of the average health level in the community was significant, it indicated that the average health level in the community could influence the mediating variable; ➂ the mediating variable was added based on step ➀; if the effect of the mediating variable was significant, and the coefficient of the average health level in the community became smaller or insignificant relative to the coefficient in step ➀, it indicated that the mediating variable had a partial or full mediating effect.

Based on the above test idea, we set the following model for testing:

The first step is to test whether the average health level in a community influences individual self-rated health.

(3) $$\begin{array}{l} {\;Health}_{i}\;={\;\alpha }_{1}\;+{\;\alpha }_{2}\bar{{\;Health}_{i}}\;+{\;\alpha }_{3}{\;X}_{i}\;+{\;\mu }_{v}\;+{\;\varepsilon }_{i} \end{array}$$

The second step was to test whether the average health level in the community influenced the mediating variable.

(4) $$\begin{array}{l} {\;Intervening\text{\_}variable}_{i}\;={\;\alpha }_{1}\;+{\;\alpha }_{2}\bar{{\;Health}_{i}}\\ \;+{\;\alpha }_{3}{\;X}_{i}\;+{\;\mu }_{v}\;+{\;\varepsilon }_{i} \end{array}$$

The third step was to add both the average health level in the community and the mediating variable into the model.

(5) $$\begin{array}{l} {\;Health}_{i}\;={\;\alpha }_{1}\;+{\;\alpha }_{2}\bar{{\;Health}_{i}}\;+{Intervening\text{\_}variable}_{i}\\ \;+{\;\alpha }_{3}{\;X}_{i}\;+{\;\mu }_{v}\;+{\;\varepsilon }_{i} \end{array}$$

where *Intervening*_*variable*_*i*_ is the mediating variable, and the remaining variables have the same meaning as in Equation (2).

## Analysis of Empirical Results

### Baseline Regression Analysis

Table 2 presents the estimates of the effect of average health levels in a community on individual self-rated health, including the estimates of the ordinary least squares (OLS) model and the corresponding cluster standard errors. In particular, column (1) lists the estimates when control variables are added; column (2) lists the estimates when health behaviour variables are controlled; column (3) lists the estimates when health behaviour variables and medical condition variables are controlled; column (4) lists the estimates when health behaviour variables, medical condition variables, and individual characteristic variables are controlled; column (5) lists the estimates when health behaviour variables, medical condition variables, individual characteristic variables, and household characteristic variables are controlled; column (6) lists the estimates when health behaviour variables, medical condition variables, individual characteristic variables, household characteristic variables, and group characteristic variables are controlled. The estimates in columns (1)–(6) show that after a series of characteristic variables were controlled, the parameter estimates of the average household health level in the community were still positive at the 1% significance level. In column (6), for example, each unit increase in the average household health level in the community contributed to a 0.718 unit increase in individual self-rated health. In conclusion, the above stepwise regression results show that an increase in the average health level in the community increased individual self-rated health, thus proving the assertion that health is contagious. This result is consistent with the conclusions of Yang and He (53) and Mueller et al. (10).

**Table 2:** OLS regression results.

**Variable**	**Explained variable: individual self-rated health**					
	**(1)**	**(2)**	**(3)**	**(4)**	**(5)**	**(6)**
avg_ health	0.7688*** (0.0147)	0.7368*** (0.0145)	0.7164*** (0.0145)	0.6472*** (0.0141)	0.6427*** (0.0141)	0.7180*** (0.0147)
Smoking		−0.0480* (0.0290)	−0.0527* (0.0289)	0.0080 (0.0295)	0.0149 (0.0294)	0.0170 (0.0292)
smoking_s		0.0008 (0.0015)	0.0014 (0.0015)	−0.0066*** (0.0015)	−0.0062*** (0.0015)	−0.0056*** (0.0014)
Exercise		0.0171*** (0.0028)	0.0183*** (0.0028)	0.0095*** (0.0028)	0.0099*** (0.0028)	0.0080*** (0.0027)
Drinking		−0.1638*** (0.0231)	−0.1650 (0.0230)	−0.2161*** (0.0226)	−0.2074*** (0.0226)	−0.2074*** (0.0225)
Insurance		0.0350*** (0.0046)	0.0443*** (0.0047)	0.0189*** (0.0048)	0.0170*** (0.0050)	0.0145*** (0.0050)
Sleep		0.0498*** (0.0024)	0.0485*** (0.0024)	0.0233*** (0.0024)	0.0232*** (0.0025)	0.0241*** (0.0025)
medical_ level			0.0813*** (0.0109)	0.0848*** (0.0105)	0.0854*** (0.0105)	0.0820*** (0.0104)
medical_ satisfaction			0.0359*** (0.0117)	0.0543*** (0.0112)	0.0555*** (0.0112)	0.0558*** (0.0111)
medical_ place			0.0513*** (0.0050)	0.0475***(0.0049)	0.0500*** (0.0049)	0.0490*** (0.0049)
Gender				0.0472** (0.0189)	0.0515*** (0.0189)	0.0672*** (0.0192)
Age				−0.0458*** (0.0025)	−0.0474*** (0.0025)	−0.0493*** (0.0025)
age2				0.0003*** (0.0000)	0.0003*** (0.0000)	0.0003*** (0.0000)
Marry				−0.0280*** (0.0094)	−0.0239*** (0.0094)	−0.0143 (0.0094)
edu				0.0325*** (0.0069)	0.0241*** (0.0071)	0.0256*** (0.0078)
Mobile				0.0402** (0.0198)	0.0236 (0.0199)	0.0214 (0.0198)
fml_count					0.0037 (0.0037)	0.0055 (0.0037)
lnfinc					0.0529*** (0.0062)	0.0583*** (0.0062)
Urban					−0.0204 (0.0172)	0.0188 (0.0177)
avg_age						0.0159*** (0.0010)
avg_lnfinc						−0.0487*** (0.0077)
avg_gender						−0.1222*** (0.0375)
avg_edu						−0.0050 (0.0136)
_cons	0.6846*** (0.0444)	1.0628*** (0.0506)	0.6010*** (0.0612)	1.7528*** (0.0934)	1.2301*** (0.1100)	0.8670*** (0.1215)
Prefecture/county dummy variable	Yes	Yes	Yes	Yes	Yes	Yes
*N*	20.371	20.371	20.371	20.371	20.371	20.371
Adj *R*^2^	0.1188	0.1462	0.1554	0.2195	0.2225	0.2330

*,**, and ***Indicate significance at the 10, 5, and 1% levels, respectively; cluster standard errors at the county and prefecture levels are reported in parentheses.

The estimates of the control variables are explained in this section. In terms of health behaviour variables, it is clear from the estimates that people who smoke and drink have worse health than those who do not. This is because the coefficient of smoking and drinking is significantly negative, and the more they smoke and drink, the worse their health become. Conversely, regular exercise has a positive effect on individual health and is positive at the 1% level of significance, which indicates that individuals who exercise regularly are healthier. However, notably, healthy individuals are also more likely to exercise. As far as our research is concerned, we do not focus on the causal relationship between the two; therefore, we will not discuss it here. Individuals with medical insurance are healthier than those without medical insurance. It can be observed from the estimates of the medical condition variables that local medical care levels, satisfaction with medical care, and hospital type all have a significant positive effect on individual health. For individual characteristic variables, household characteristic variables, and group characteristic variables, men, individuals with higher educational levels, and individuals with higher household income levels also have a significant positive effect on individual household health. Finally, given the potential endogeneity issues with some of the control variables, we cannot interpret or derive much from the results and economic implications of these variables.

### Endogeneity Control

Notably, relying only on baseline OLS regressions may result in potential endogeneity issues. The endogeneity stems from two main sources. On the one hand, there is a potential problem of omitted variables in the model, as there are numerous observable and unobservable factors affecting individual health. Even for observable factors, this study cannot encompass all influences, much less for unobservable factors. The problem of omitted variables may have biassed estimates. On the other hand, reverse causality may exist in the baseline model (i.e., the health status of an individual affects the health level of the group). This is because the health behaviour of an individual affects the health behaviour of other individuals, which makes up the health level of the group other than the household. Therefore, such reverse causality exists. This possible reverse causality would mean that the effect of average health levels in a community on individual self-rated health is overestimated.

Several measures were taken to address these two issues. First, we included as many control variables as possible (i.e., health behaviour variables, medical condition variables, individual characteristic variables of the householder, household characteristic variables, group characteristic variables, and a dummy variable for prefecture or county). Second, drawing on Yu and Zhan (43), we used the average well-being of other households living in the same community with the respondent household as an instrumental variable for the average health level of the respondent community and performed a regression using a two-stage least squares (2SLS) model. This measure is reasonable because the average well-being of other households living in the same community with the respondent household reflects the average level of well-being of the residents in the area; it is closely related to the health level of households other than the respondent household. In general, the higher the average health level in the community, the higher the average well-being. Alternatively, the higher the average well-being of the community, the higher its average health. Therefore, we can be sure that there is a correlation between the average health level in the community and the average well-being in the community, satisfying the correlation condition of the instrumental variable. Conversely, the well-being of other households is not directly correlated with the health of the respondent household, thereby satisfying the exogeneity condition of the instrumental variable.

Table 3 presents the estimates of the 2SLS model for instrumental variables. The results of Stage I regression show that the average health in the community was significantly positively correlated with the average well-being in the community, which was consistent with the conclusions of Christakis and Fowler (27) and Polsky et al. (65). Moreover, the F value of the weak instrumental variable was much >10, indicating that the selected instrumental variable was highly correlated with the endogenous explanatory variables. Therefore, the possibility of a weak instrumental variable can be excluded. The results of the Stage II regression show that the coefficients of the average health in a community were all positive (at least at the 1% level of significance), and this remained consistent in the regression results of the baseline model. This indicates that the contagion effect of health remained significant after potential endogeneity issues were overcome using the instrumental variable. Therefore, the average health in the community has a significant effect on individual self-rated health, which implies that health is contagious.

**Table 3:** 2SLS regression results for instrumental variables.

**Variable**	**Explained variable: individual self-rated health**				
	**(1)**	**(2)**	**(3)**	**(4)**	**(5)**
avg_health	0.8669^***^ (0.0618)	0.8248^***^ (0.0631)	0.8646^***^ (0.0597)	0.8579^***^ (0.0595)	0.7930^***^ (0.0546)
Health behaviour variables	Yes	Yes	Yes	Yes	Yes
Medical condition variables	No	Yes	Yes	Yes	Yes
Individual characteristic variables	No	No	Yes	Yes	Yes
Household characteristic variables	No	No	No	Yes	Yes
Group characteristic variables	No	No	No	No	Yes
Prefecture/county dummy variable	Yes	Yes	Yes	Yes	Yes
	**Stage I regression**				
Average well-being in the community	0.1294^***^ (0.0037)	0.1263^***^ (0.0037)	0.1290^***^ (0.0037)	0.1294^***^ (0.0037)	0.1398^***^ (0.0035)
Stage I F value	188.97	153.06	124.43	107.51	203.82
*N*	20,371	20,371	20,371	20,371	20,371
Adj *R*^2^	0.0687	0.0759	0.0934	0.0947	0.1929

*** *Indicate significance at the 1% levels; cluster standard errors at the county and prefecture levels are reported in parentheses*.

### Robustness Tests

The above regression analysis reveals that the average health level in a community has a significant positive effect on individual self-rated health, supporting the assertion that health is contagious. However, this finding needs to be verified with caution. To further test the reliability of the baseline model, we applied two different strategies to analyse the estimation results of the baseline model.

### Robustness Test Based on Objective Health Indicators

The health indicator we used in the baseline regressions was respondents' self-rated health, which was provided by respondents based on their responses to health questions. Subsequently, there was a problem with biassed answers, as respondents may have provided answers that did not match their actual health status. For example, if respondents suffer from a certain disease and may not want others to know about their condition for privacy protection, they may provide answers that do not match the reality, resulting in biassed answers and thus affecting the estimation results. To overcome this problem, we replaced the original subjective self-rated health indicators with objective health indicators for the regression analysis. Referring to existing literature, we selected two variables to represent the objective health indicators: “Were you hospitalised due to illness in the past year?” and “total medical expenses.” Table 4 presents the results of the estimation. The estimates in columns (1) and (2) show that the parameter estimates of the average health level were significantly negative after all variables were controlled in turn, which coincided with our expectations. However, the sign was the opposite of the baseline results, as the baseline regression analysis was based on subjective self-rated health with a positive sign. Conversely, objective health indicators reflect actual conditions, and higher average health levels have a negative impact on existing objective health. Hence, the sign was the opposite, which also indirectly verified the robustness of the estimation results of the baseline regression model.

**Table 4:** Robustness test based on objective health indicators.

**Variable**	**Were you hospitalised due to illness in the past year?**	**Total medical expenses**
	**(1)**	**(2)**
avg_health	−0.0512^***^ (0.0047)	−2016.425^***^ (223.6289)
Health behaviour variables	Yes	Yes
Medical condition variables	Yes	Yes
Individual characteristic variables	Yes	Yes
Household characteristic variables	Yes	Yes
Group characteristic variables	Yes	Yes
Prefecture/county dummy variable	Yes	Yes
*N*	20,371	20,371
Adj *R*^2^	0.0908	0.0484

*** *Indicate significance at the 1% levels; cluster standard errors at the county and prefecture levels are reported in parentheses*.

### Robustness Tests Based on Different Estimation Methods

We used an OLS model in the baseline regression because the dependent variable in this study was taken in an ordered manner, and using an OLS model enabled a better estimation of the marginal effects through which effect size could be compared. In this section, we re-estimate the baseline model using both the ordered probit model and the ordered logit model. Notably, that the estimates here were not marginal effects but original coefficients and did not affect our analysis of the results. Our focus was on the direction of the coefficients rather than on their comparability and magnitude. Based on this logic, we re-estimated the baseline model using the two estimation methods described above. Table 5 reports the results of the estimations. The results show that the average health level in the community still had a significant positive effect on individual self-rated health, thus providing further evidence for the assertion that health is contagious. Therefore, the estimation results of the baseline model are considered reliable.

**Table 5:** Robustness test for the regression based on ordered probit and ordered logit.

**Variable**	**Ordered probit**	**Ordered logit**
	**(1)**	**(2)**
Avg_health	0.7569^***^ (0.0160)	1.3481^***^ (0.0280)
Health behaviour variables	Yes	Yes
Medical condition variables	Yes	Yes
Individual characteristic variables	Yes	Yes
Household characteristic variables	Yes	Yes
Group characteristic variables	Yes	Yes
Prefecture/county dummy variable	Yes	Yes
*N*	20,371	20,371
Pseudo *R*^2^	0.0911	0.0944

*** *Indicate significance at the 1% levels; cluster standard errors at the county and prefecture levels are reported in parentheses*.

### Heterogeneity Analysis

#### Heterogeneity Between Urban and Rural Areas and Among Eastern, Central, and Western Regions

Owing to the unique urban-rural dual system and the uneven regional development in China, urban/rural areas and eastern, central, and western regions should be treated in different ways when analysing whether health is contagious. In this section, we explore whether health contagion exhibits different characteristics depending on the urban/rural and regional affiliations of households. For this purpose, we divided the study samples into urban and rural samples according to the urban and rural areas to which the samples belong and eastern, central, and western samples according to the regions where the samples are located. Table 6 presents the estimation results. Based on the estimation results for urban and rural areas, we observed that the coefficient of average health level was positive at the 1% significance level for both urban and rural samples, which indicates that health contagion exists in both urban and rural areas. However, the coefficient for rural samples was greater than that for urban samples (0.7068 > 0.2587), indicating that the contagion effect of health is more pronounced in rural areas. From the estimated results for different regions, we found that the estimated coefficients for the average health level in the eastern, central, and western regions were all significantly positive, which indicates that the contagion effect of health is also significant in all regions. Nevertheless, the coefficient values for the central, eastern, and western regions were ranked as 0.7229 > 0.7152 > 0.7047, respectively. This suggests that the contagion effect of health was strongest in the central region, followed by the eastern region. The contagion effect of health was weakest in the western region. Overall, the contagion effect of health was more pronounced in rural areas of the central region.

**Table 6:** Regression results for health contagion among urban and rural areas and different regions.

**Variable**	**Explained variable: individual self-rated health**				
	**Urban areas**	**Rural areas**	**Eastern region**	**Central region**	**Western region**
	**(1)**	**(2)**	**(3)**	**(4)**	**(5)**
Avg_health	0.2587^***^ (0.0173)	0.7068^***^ (0.0254)	0.7152^***^ (0.0215)	0.7229^***^ (0.0273)	0.7047^***^ (0.0304)
Health behaviour variables	Yes	Yes	Yes	Yes	Yes
Medical condition variables	Yes	Yes	Yes	Yes	Yes
Individual characteristic variables	Yes	Yes	Yes	Yes	Yes
Household characteristic variables	Yes	Yes	Yes	Yes	Yes
Group characteristic variables	Yes	Yes	Yes	Yes	Yes
Prefecture/county dummy variable	Yes	Yes	Yes	Yes	Yes
*N*	10,634	9,737	8,508	6,000	5,863
Adj *R*^2^	0.2587	0.2181	0.2393	0.2282	0.2368

*** *Indicate significance at the 1% levels; cluster standard errors at the county and prefecture levels are reported in parentheses*.

#### Heterogeneity by Gender and Medical Care Level

A new study from Macquarie University in Australia found that an unhealthy body type with low fat content is attractive to women; however, for men, a healthy body type with normal body fat content is more attractive. This study aimed to show that men and women have different views of health. Accordingly, health contagion is also likely to reveal differences between men and women. To test this assertion, we divided the study samples into males and females and ran separate regressions to corroborate our assertion. The level of medical care is also an important factor that influences health. In areas with high levels of medical care, the contagion effect of health should be stronger, and people should be more concerned about their health status. Therefore, we also divided the study samples according to the level of medical care and ran separate regressions. Table 7 reports the results of the estimations. Columns (1) and (2) are estimates based on the level of medical care; columns (3) and (4) are estimates based on gender. The results in columns (1) and (2) show that while the average health level had a significant effect in both areas with high and low levels of medical care, the contagion effect of health was more pronounced in areas with low levels of medical care (0.7339 > 0.6971). This was not consistent with our previous assertion. This is because regardless of the high or low level of medical care, people are more concerned about their health status—the concept of health is deeply rooted in their minds. In rural areas, medical supply fails to meet people's medical needs because of the limited medical level. When people suffer from major diseases, they choose to go to better hospitals in cities. Therefore, the contagion effect of health is more obvious in rural areas. From the results in columns (3) and (4), we can see that the contagion effect of health is significant among both males and females, but there is no significant difference between them (0.7169 vs. 0.7180). This indicates that although men and women have different views of health, both are concerned about their health, and there is no real difference in the contagion effect of health.

**Table 7:** Regression results for health contagion based on differences in medical care level and gender.

**Variable**	**Explained variable: individual self-rated health**			
	**High level of medical care**	**Low level of medical care**	**Male**	**Female**
	**(1)**	**(2)**	**(3)**	**(4)**
Avg_health	0.6971^***^ (0.0205)	0.7339^***^ (0.0209)	0.7169^***^ (0.0198)	0.7180^***^ (0.0218)
Health behaviour variables	Yes	Yes	Yes	Yes
Medical condition variables	Yes	Yes	Yes	Yes
Individual characteristic variables	Yes	Yes	Yes	Yes
Household characteristic variables	Yes	Yes	Yes	Yes
Group characteristic variables	Yes	Yes	Yes	Yes
Prefecture/county dummy variable	Yes	Yes	Yes	Yes
*N*	11,078	9,293	10,475	9,896
Adj *R*^2^	0.2210	0.2336	0.2376	0.2233

*** *Indicate significance at the 1% levels; cluster standard errors at the county and prefecture levels are reported in parentheses*.

#### Analysis by Age

The infectivity of health also shows heterogeneity among different age groups. Generally, compared with teenagers and middle-aged groups, older people focus more on their health and medical care information. To test whether the average health level of the community shows significant differences due to different age groups, we divide the total sample into three sub-samples—under 18 years old, 18–60 years old, and over 60 years old—and perform regressions. The results are listed in Table 8. From the estimation results, we found that the parameter estimates of the average health level of the community were significant in the 18–60 and 60-year-old groups, which indicates that healthy infectiousness exists in these two groups. However, judging from the size of the coefficient, the contagion effect of people over 60 years of age is more obvious.

**Table 8:** Regression results for different age groups of healthy infectiousness.

**Variable**	**Explained variable: Individual self-rated health**		
	**Under 18**	**18–60 years old**	**Over 60 years old**
	**(1)**	**(2)**	**(3)**
Avg_health	0.5320 (0.6809)	0.4191* (0.2490)	0.5476** (0.2556)
Health behaviour variables	Yes	Yes	Yes
Medical condition variables	Yes	Yes	Yes
Individual characteristic variables	Yes	Yes	Yes
Household characteristic variables	Yes	Yes	Yes
Group characteristic variables	Yes	Yes	Yes
Prefecture/county dummy variable	Yes	Yes	Yes
*N*	736	13,290	6,345
Adj *R*^2^	0.2087	0.2534	0.2107

* and **Indicate significance at the 10 and 5 levels, respectively; cluster standard errors at the county and prefecture levels are reported in parentheses.

## Mechanism Testing

### Testing Health Learning or the Imitation Mechanism

The health learning or imitation mechanism refers to a person learning from or imitating the positive health behaviour of others or making observations of the behaviours of members of the reference group to change their own health status. The test results of the mediating effect model are presented in Table 9. Column (1) lists the test results of the first step, which indicates that the average health level in the community significantly improved the self-rated health of individuals. Column (2) shows the test results of the second step, which indicate that the average health level in the community had a significant effect on the learning or imitation mechanism. Column (3) presents the test results of the third step, which show that the effect of the learning or imitation mechanism on individual self-rated health was significant, thus proving the existence of a mediating effect. Additionally, when the mediating variables were controlled, the coefficients of the average health level in the community decreased compared to those in the baseline regression. Therefore, we concluded that the learning or imitation mechanism had a partial mediating effect (15.72%).

**Table 9:** Test results for the learning or imitation mechanism of health contagion.

**Variable**	**Individual self-rated health**	**Learning/imitation mechanism**	**Individual self-rated health**
	**(1)**	**(2)**	**(3)**
Avg_health	0.7059*** (0.0146)	0.6322*** (0.0047)	0.6802*** (0.0167)
Learning/imitation mechanism			0.1408* (0.0755)
Health behaviour variables	Yes	Yes	Yes
Medical condition variables	Yes	Yes	Yes
Individual characteristic variables	Yes	Yes	Yes
Household characteristic variables	Yes	Yes	Yes
Group characteristic variables	Yes	Yes	Yes
Prefecture/county dummy variable	Yes	Yes	Yes
*N*	20,371	20,371	20,371
Adj *R*^2^	0.2229	0.5196	0.2356
Mechanism identification	The mechanism is effective.		

* and ***Indicate significance at the 10 and 1% levels, respectively; cluster standard errors at the county and prefecture levels are reported in parentheses.

### Testing the Social Interaction Mechanism of Health Contagion

The social interaction mechanism of health contagion refers to an individual being able to obtain relevant information about health through direct communication and discussion with members of the reference group. We used “favour spending” from the CFPS2018 database as a proxy variable for the social interaction mechanism and tested this mechanism using a mediating effect model. The test results are presented in Table 10. The results show that the social interaction mechanism of health contagion was significant because the regression coefficients in columns (2) and (3) of Table 10 were significant at the 1% level. Thus, health is contagious through the social interaction mechanism, although the contagion effect is relatively small (6.95e-06^*^0.7246/393.34).

**Table 10:** Test results on the social interaction mechanism of health contagion.

**Variable**	**Individual self-rated health**	**Social interaction mechanism**	**Individual self-rated health**
	**(1)**	**(2)**	**(3)**
Avg_health	0.7246^***^ (0.0152)	393.3439^***^ (94.1103)	0.7219^***^ (0.0152)
Social interaction mechanism			6.95e−06^***^ (1.13e−06)
Health behaviour variables	Yes	Yes	Yes
Medical condition variables	Yes	Yes	Yes
Individual characteristic variables	Yes	Yes	Yes
Household characteristic variables	Yes	Yes	Yes
Group characteristic variables	Yes	Yes	Yes
Prefecture/county dummy variable	Yes	Yes	Yes
*N*	20,371	20,371	20,371
Adj *R*^2^	0.1557	0.0418	0.1572
Mechanism identification	The mechanism is effective.		

*** *Indicate significance at the 1% levels; cluster standard errors at the county and prefecture levels are reported in parentheses*.

## Limitations and Future Research Directions

This paper focused on the impact of community average health level on individual self-rated health, and used instrumental variable method to deal with its endogeneity, but the problem of endogeneity hasn't been completely solved. Since our study topic is whether health is contagious, we emphasise the neighbourhood effect of health, that is, the neighbourhood effect of health is used to characterise the infectivity of health. According to the existing research, we calculated the average self-rated health level of the community, used the average self-rated health level of the community as the proxy variable of neighbourhood effect, and took it as the core explanatory variable. Our endogenous treatment is also based on the causal relationship between the average health level of the community and individual self-rated health. However, we also added some health behaviour variables, such as whether to smoke, the amount of smoking, the frequency of exercise, and so on. If only these variables are added into the model as control variables, there will be inevitably causal relationship between them and individual self-rated health. These variables as health habit variables will affect individual self-rated health, on the contrary, individual self-rated health will also affect health habit variables. This paper didn't solve the endogenous problem between health habit variables and individual self-rated health. In the future, we will further explore the impact of health habit variables on individual self-rated health, focusing on the causal relationship between health habits and individual self-rated health, which is the main content of our next paper.

## Conclusions

Based on the CFPS 2018 data, we examined whether health was contagious. We also employed an instrumental variable approach to mitigate the endogeneity caused by measurement error and reverse causation and tested the mechanisms of underlying health contagion. We found that after controlling for health behaviour variables, medical condition variables, individual characteristic variables, household characteristic variables, group characteristic variables, and prefecture/county characteristic variables, the parameter estimates for the average health level in the community were positive at the 1% significance level, supporting the assertion that health is contagious. Quantitatively, each unit increase in the average household health level in the community would contribute to a 0.718 unit increase in individual self-rated health. To control for endogeneity, we used the average well-being of other households living in the same community (excluding the respondent household) as an instrumental variable for the average health level in the respondent community, which was consistent with the baseline model. In addition, robustness tests were performed using two different strategies, which yielded results consistent with the baseline model. Furthermore, the heterogeneity analysis showed that the contagion effect of health differed depending on the level of medical care, regional affiliation, gender, rural/urban, and age groups. Specifically, the contagion effect of health was more pronounced in the elderly population and the central rural areas, where the level of medical care was lower, while the difference between genders was not significant. Further research found that the contagion of health was achieved through learning or imitation mechanisms and social interaction mechanisms.

## Data Availability Statement

The original contributions presented in the study are included in the article/supplementary material, further inquiries can be directed to the corresponding authors.

## Author Contributions

All authors undertook research, writing, review tasks throughout this study, read, and agreed to the published version of the manuscript.

## Conflict of Interest

The authors declare that the research was conducted in the absence of any commercial or financial relationships that could be construed as a potential conflict of interest.

## Footnotes

^1^The frequency is in weeks.

^2^There are four types of medical insurance: urban residents' medical insurance, new rural cooperative medical insurance, publicly funded medical insurance, and supplementary medical insurance.

^3^Very dissatisfied is 1, dissatisfied is 2, generally is 3, relatively satisfied is 4, very satisfied is 5.
